# Difference in trends in suicide mortality rates by marital status and age groups from 2000 to 2020 in Japan

**DOI:** 10.1371/journal.pone.0342843

**Published:** 2026-03-31

**Authors:** Tasuku Okui

**Affiliations:** Medical Information Center, Kyushu University Hospital, Fukuoka City, Japan; PUCRS: Pontificia Universidade Catolica do Rio Grande do Sul, BRAZIL

## Abstract

This study examined trends in suicide mortality rates by marital status across age groups in Japan, which have not been investigated to date. We utilized the Vital Statistics and Census data of Japan, which cover nationwide suicide mortality and population. Marital status was categorized as married, never-married, widowed, and divorced, while age groups were divided into 20–39 years, 40–64 years, and ≥65 years. Age-standardized mortality rates (ASMRs) of suicide were calculated by sex, marital status, and each age group. Annual percent changes in suicide mortality rates between 2000 and 2020 were also determined by age-adjusted Poisson regression analysis, followed by another regression analysis evaluating interactions between marital status and year effects by age group. Moreover, an age–period–cohort analysis by marital status was conducted to determine birth cohort effects. In this period, a significant declining trend in suicide mortality rate was observed across all marital status groups among persons aged 40–64 years and ≥65 years, whereas significant downward trends were not observed for never-married men aged 20–39 years and widowed and divorced women aged 20–39 years. Additionally, the interaction between never-married persons and year was significantly positive relative to married persons among men aged 20–39 years. Moreover, the rate ratios of ASMRs for widowed and divorced persons relative to those for married persons tended to be higher among younger persons across the study period. The results of the age–period–cohort analysis revealed that mortality risk increased in recently born cohorts, particularly among widowed and divorced persons, for women. Overall, trends in suicide mortality rates by marital status differed according to age group. Additionally, the suicide mortality rate for divorced and widowed persons aged 20–39 years remained particularly high, suggesting a possible need for targeted suicide prevention efforts in this population.

## Introduction

Suicide is a major cause of death that also has a profound impact on people close to the those who died by suicide. Globally, suicide mortality rates decreased in many countries between 2000 and 2019 [1], while the trends in the rates differed depending on regions [2]. In Japan, the age-standardized mortality rate (ASMR) of suicide decreased during the 2010s [3–5], although it showed an increased trend during the COVID-19 pandemic periods. Several socioeconomic factors, including employment status, educational attainment, urbanization level, and regional income, are known to be associated with suicide risks in Japan [5–7].

The suicide mortality rate also varies by marital status. Numerous studies conducted in Japan and other countries have shown that married persons tend to have a lower risk of suicide compared to those with other marital statuses [8–12], even though the proportion of married persons is considered to differ depending on countries. It was also shown in the United Sates that being married was associated with a lower risk of suicidal ideation [13]. Moreover, studies in South Korea, Hong Kong, and the United States have indicated that the association between marital status and suicide risk can vary by age group [8,9,14]. In Japan, the trends in the ASMRs of suicide among persons aged 40 years or older have been reported [15]. In contrast, the proportion of each marital status largely changes depending on age groups in Japan, and the characteristics of each marital status is considered to also vary depending on age groups [16,17]. Therefore, it is important to investigate the association between marital status and suicide mortality rate by age groups. The White Paper on Suicide Countermeasures, published by the Ministry of Health, Labour and Welfare, presented suicide mortality rates by combinations of age group and marital status [18], showing substantial variation in these associations depending on age. In contrast, previous studies in Japan have not investigated trends in suicide rates by marital status across age groups. Therefore, it remains unclear how suicide mortality rates have changed over the decades within specific subpopulations. A study from Hong Kong revealed that the trends by marital status differed across age groups [8], suggesting that such trends were age-dependent. Investigating these patterns is important to identify specific subpopulations in need of focused suicide prevention efforts. Moreover, it is meaningful to investigate birth cohort effects on suicide mortality rates by marital status to understand the differences in suicide mortality rates by marital status given that birth cohort influences suicide mortality rates in Japan [19,20].

In this study, we investigated trends in suicide mortality rates by marital status using national data from Japan.

## Materials and methods

We used data from Japan’s Vital Statistics to obtain nationwide suicide mortality data [21]. These data included all recorded suicide deaths among Japanese persons that occurred in Japan. Suicide mortality data from 2000 to 2020 were extracted by sex, age group, and marital status. The Japanese population data every five years from 2000 to 2020 were obtained from the Census of Japan [22]. Because the Census, which surveys population by marital status, is conducted every 5 years, population in the years when the Census was not conducted was estimated by linear interpolation. Specifically, a linear regression model using population and year as outcome and explanatory variables, respectively, was applied to the data by sex, age group, and marital status. The data were accessed on February 8^th^ 2025. The author did not have access to information that could identify individual participants during or after data collection.

Age groups in the Census data were defined as follows: 20–24 years, 25–29 years, 30–34 years, 35–39 years, 40–44 years, 45–49 years, 50–54 years, 55–59 years, 60–64 years, 65–69 years, 70–74 years, 75–79 years, 80–84 years, and ≥85 years. In order to align with the Census data, suicide mortality cases aged 85 years and older were grouped into a single category. Marital status was categorized as married, never-married, widowed, or divorced. For analysis, we further grouped ages into three categories: 20–39 years, 40–64 years, and ≥65 years to represent young, middle-aged, and older persons, respectively.

We calculated the proportion of each marital status by sex and year for 20–39, 40–64, and ≥65 age groups. The ASMRs of suicide were calculated by sex, marital status, and year for each age group. The direct standardization method was used for the calculation of the ASMR [23]. Specifically, the population in each five-year age group of the standard population was multiplied by the corresponding five-year age group-specific suicide mortality rate of the population of interest. Using this procedure, the expected number of suicides for each five-year age group was obtained. The ASMR for the population of interest was then calculated by dividing the sum of the expected number of suicides across all five-year age groups by the total standard population. Age-specific 2020 population data were used as the standard population for the respective age groups. The 95% confidence interval (CI) for the ASMR was calculated by assuming that the number of suicides follow the Poisson distribution [24]. We also calculated the ratios of the ASMRs for never-married, widowed, and divorced persons compared to married persons, stratified by sex, year, and age groups. The 95% CI for the ratio of the ASMR was calculated by the delta method.

Furthermore, annual percent changes (APCs) in suicide mortality rates between 2000 and 2020 were calculated by age-adjusted Poisson regression analysis. Specifically, the number of suicides was used as the outcome variable, and five-year age groups and year were used as the explanatory variables. Logarithm of population was used as the offset term, and the analysis was conducted by sex, marital status, and the age groups (20–39, 40–64, and ≥65 age groups). An APC, 95% CI, and p-value were calculated by sex, marital status, and the age groups, and p-value of <0.05 was judged as statistically significant. In addition, Poisson regression analysis was conducted using five-year age groups, marital status, year, and an interaction term between marital status and year according to sex and age groups (i.e., 20–39, 40–64, and ≥65 years) to reveal and test differences in trends by marital status. Rate ratios (RR), 95% CIs, and *p*-values were calculated. Newey–West standard errors were calculated in the Poisson regression analyses to address autocorrelation in the observations.

Moreover, age–period–cohort analysis was conducted by marital status and sex to distinguish the effects of age, period, and birth cohort on suicide mortality risk according to marital status. Birth cohort was derived from year and age group. A Bayesian age–period–cohort model based on a binomial logit model was used as the model [25]. In addition to the effects of age, period, and birth cohort, a heterogeneity term was included as an explanatory variable to address overdispersion [25]. The sums of age, period, and cohort effects were constrained to zero, and first-order random-walk priors were specified for the age, period, and birth cohort effects [25]. An inverse gamma distribution with the scale and shape parameters of 1 and 0.01, respectively, was used as the hyper prior for the precision of the random walk prior of each effect. The total number of iterations was set to 200,000, with 50,000 iterations used as burn-in. Mortality RRs and 95% credible intervals were calculated by age, period, and cohort effects. Persons aged 85 years or older were excluded from the age–period–cohort analysis, because their birth cohorts could not be identified.

All statistical analyses were conducted using R version 4.5.0 [26], with the packages bamp, ggplot2, ggpubr, lmtest, and reshape2, sandwich, and stringr used [27–32]. This study did not require ethical approval, as it relied entirely on publicly available data. In addition, this study was conducted on the basis of the Ethical Guidelines for Medical and Biological Research Involving Human Subjects in Japan [33].

## Results

**Fig 1:**
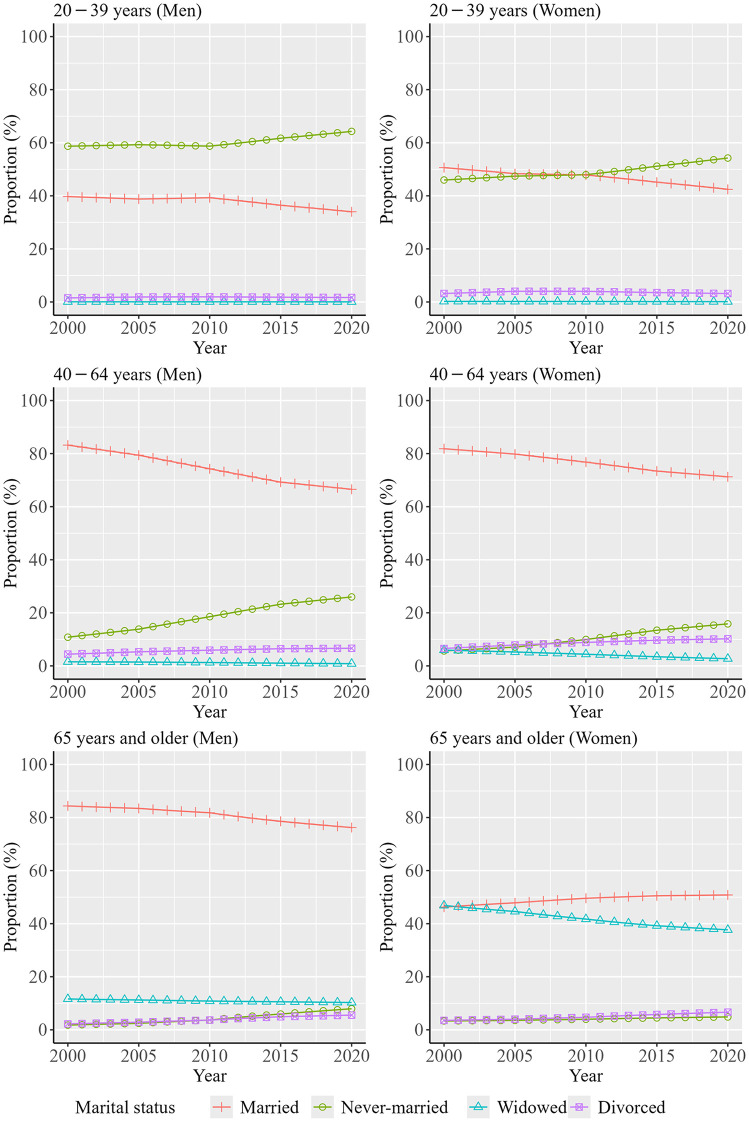
Trends in the distribution of marital status by sex and age group over the decades.

Fig 1 shows the trends in the distribution of marital status by sex and age group over the decades. Data from Fig 1 are shown in S1 Table. The proportion of married persons tended to decline from 2000 to 2020, whereas the proportion of never-married persons increased across all age groups during the same period. Additionally, the proportion of never-married persons was highest among persons aged 20–39 years from 2010, while the proportion of married persons was highest among those aged 40 − 64 and ≥65 years.

**Fig 2:**
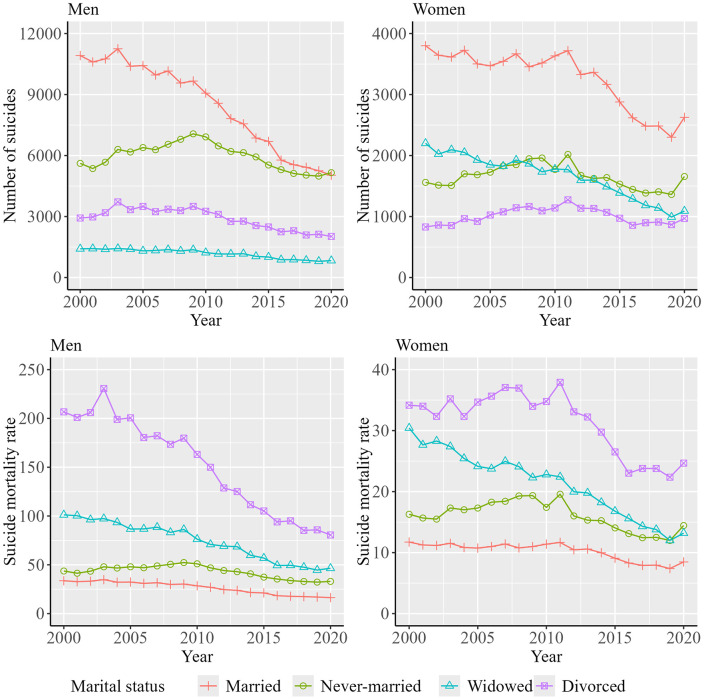
Trends in the number of suicides and suicide mortality rate by sex and marital status over the decades. The suicide mortality rate indicates the rate per 100,000 persons.

Fig 2 shows the trends in the number of suicides by sex, age group, and marital status over the decades. Data from Fig 2 are shown in S2 Table. The number of suicides was the largest among married persons in men and women, while the crude suicide mortality rate was the largest among divorced persons.

**Fig 3:**
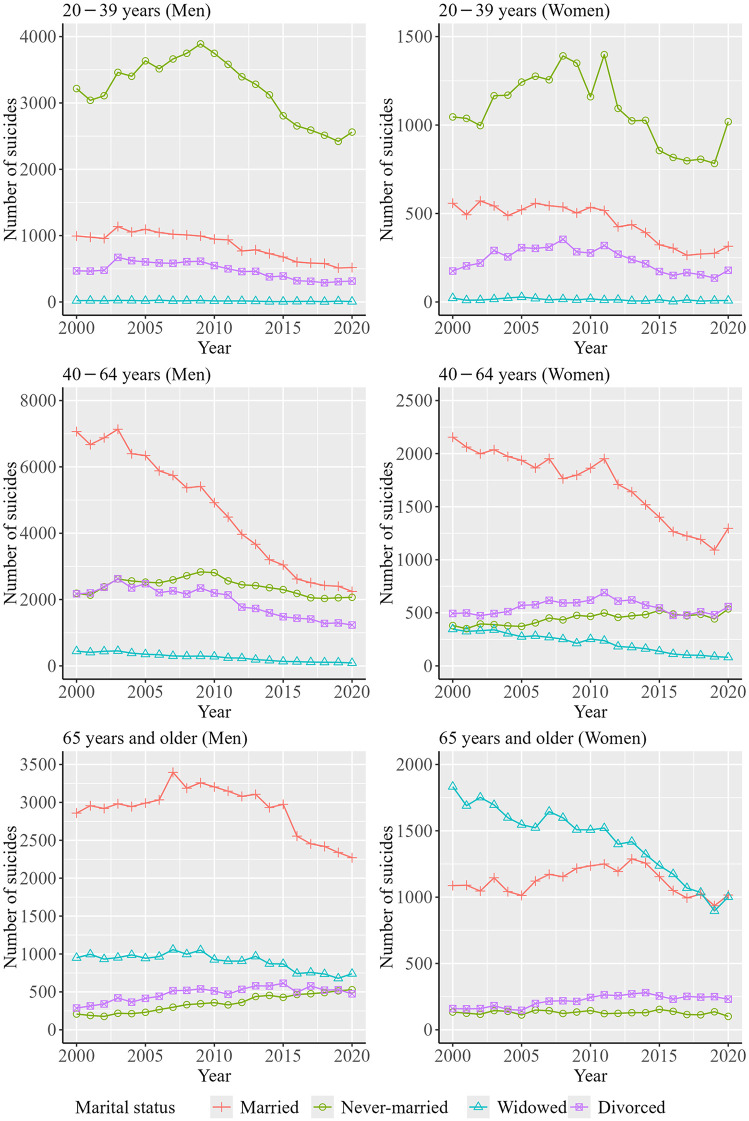
Trends in the number of suicides by sex, age group, and marital status over the decades.

Fig 3 shows the trends in the number of suicides by sex, age group, and marital status over the decades. Data from Fig 3 are shown in S3 Table. The number of suicides for married persons was the largest among persons aged 40 − 64 years, while that for never-married persons was the largest among persons aged 20–39 years. Among persons aged ≥65 years, the number of suicides was the largest in married persons and widowed persons for men and women, respectively. In addition, the degree of decrease in the number of suicides among men aged ≥65 years and men and women aged 40 − 64 years was the largest in married persons, while that among women aged ≥65 years was the largest in widowed persons.

**Fig 4:**
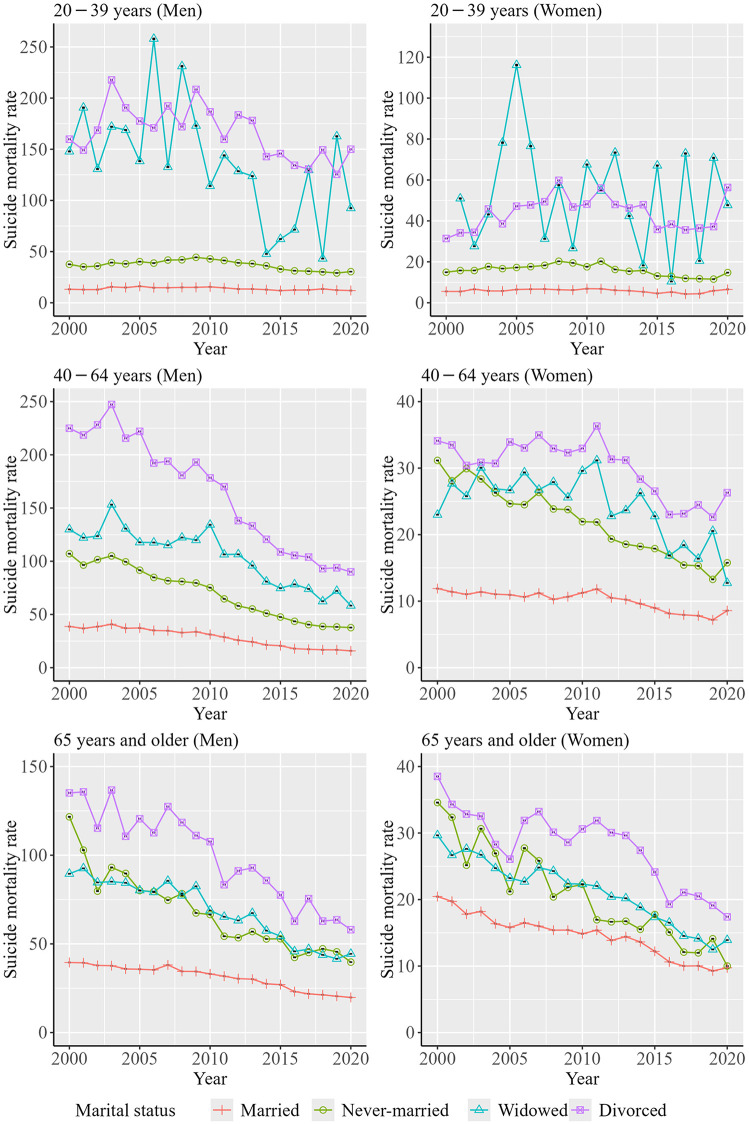
Trends in the ASMRs by sex, age group, and marital status. Suicide mortality rate indicates the ASMR of suicide per 100,000 persons, which was estimated by the direct standardization method.

Fig 4 presents the trends in ASMRs by sex, age group, and marital status. Data from Fig 4 are shown in S4 Table. Among persons aged 20–39 years, ASMRs for widowed or divorced persons were the highest. In addition, among men aged 20–39 years, decreasing trends in ASMRs were observed for both widowed and divorced men. For persons aged 40 − 64 and ≥65 years, ASMRs showed a decreasing trend across all marital status groups. Furthermore, among persons aged 45–64 and ≥65 years, the ASMRs for married persons were consistently the lowest, while those for divorced persons were the highest throughout the study period.

**Fig 5:**
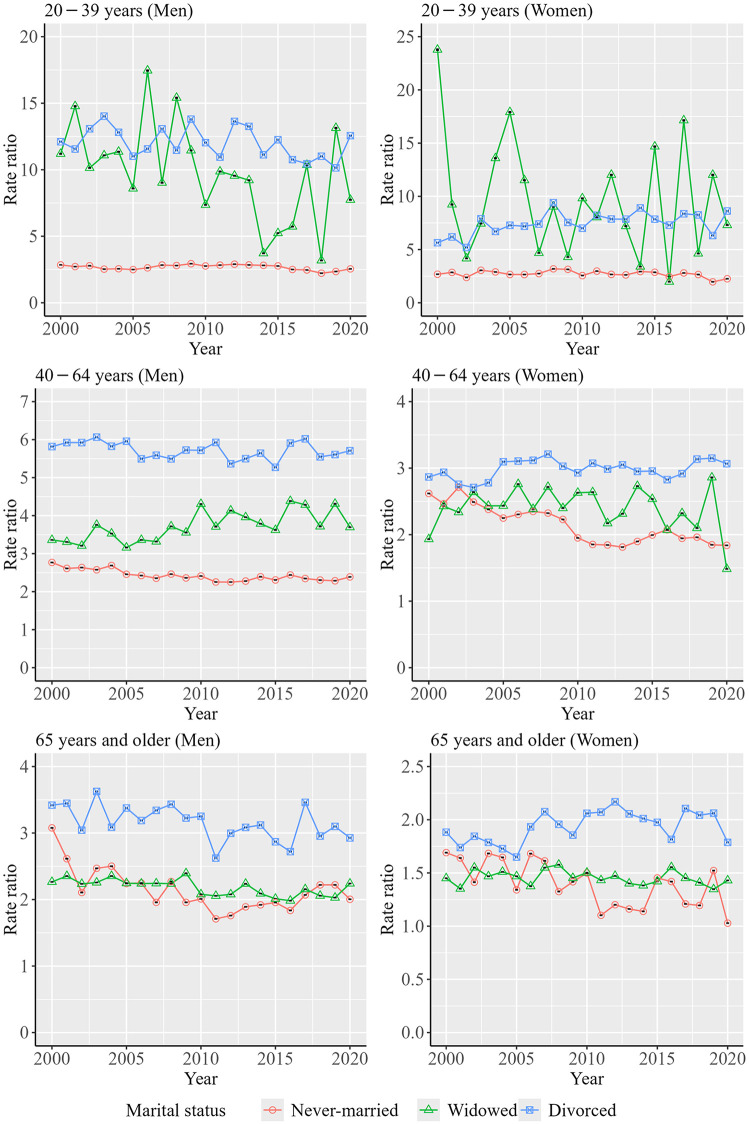
Trends in rate ratios of the ASMRs for never-married, widowed, and divorced persons relative to that of married persons, grouped by sex and age group.

Fig 5 shows the trends in rate ratios of the ASMRs for never-married, widowed, and divorced persons relative to that of married persons, grouped by sex and age group. Data from Fig 5 are shown in S5 Table. These rate ratios were consistently greater than 1 among persons aged 20–39, 40–64, and ≥65 years, indicating an increased suicide risk among never-married, widowed, and divorced persons. Over the decades, the rate ratios for widowed and divorced persons tended to be higher among those aged 20–39 years than among those aged 40–64 and ≥65. In particular, the rate ratios remained high over time among widowed and divorced persons aged 20–39 years. Additionally, the rate ratios for divorced men were consistently higher than those for divorced women. Similarly, the rate ratios for widowed men were consistently higher than those for widowed women among persons aged 40–64 and ≥65 years. Overall, the rate ratios of the ASMRs for widowed and divorced persons relative to that of married persons tended to be higher among younger age groups over the decades.

Table 1 shows the APCs in suicide mortality rates from 2000 to 2020 by sex, age group, and marital status based on age-adjusted Poisson regression model. Among persons aged 20–39 years, the largest decrease in suicide mortality rate was observed in widowed persons in men and married persons in women. The APCs for widowed persons in men and married persons in women were –3.09 (95% CI: –4.90, –1.25) and –1.36 (95% CI:–2.18, –0.54), respectively, with a statistically significant decreasing trend. In contrast, significant decreasing trends in suicide mortality rates were not observed among never-married men and widowed and divorced women. Among persons aged 40–64 and ≥65 years, a significant decreasing trend in suicide mortality rates was observed across all marital status groups for both sexes. The largest declines were found in never-married persons. Specifically, the APCs for never-married men aged 40–64 and ≥65 years were –5.41 (95% CI:–6.01, –4.80) and –5.17 (95% CI:–5.67, –4.66), respectively. For never-married women in the same age groups, the APCs were –3.66 (95% CI:–4.00, –3.33) and −4.78 (95% CI: −5.48, −4.08), respectively. Overall, suicide mortality rates showed decreasing trends in most of the cases, while a significant decreasing trend was not observed in some marital statuses among persons aged 20–39 years.

**Table 1:** APCs in suicide mortality rates from 2000 to 2020 by sex, age group, and marital status based on age-adjusted Poisson regression model.

Sex and age group	Married	Never–married	Widowed	Divorced
	APC (95% CI)	APC (95% CI)	APC (95% CI)	APC (95% CI)
Men				
20 − 39 years	–1.61*** (–2.39, –0.82)	–0.69 (–1.61, 0.24)	–3.09** (–4.90, –1.25)	–2.08*** (–2.99, –1.16)
40 − 64 years	–4.71*** (–5.53, –3.87)	–5.41*** (–6.01, –4.80)	–4.69*** (–5.56, –3.80)	–5.32*** (–6.06, –4.58)
≥65 years	–3.51*** (–4.09, –2.93)	–5.17*** (–5.67, –4.66)	–3.78*** (–4.29, –3.27)	–4.93*** (–5.61, –4.24)
Women				
20 − 39 years	–1.36** (–2.18, –0.54)	–1.09* (–2.16, –0.01)	–0.79 (–3.38, 1.88)	–0.27 (–1.59, 1.06)
40 − 64 years	–2.00*** (–2.52, –1.47)	–3.66*** (–4.00, –3.33)	–2.77*** (–3.47, –2.06)	–1.86*** (–2.50, –1.22)
≥65 years	–3.21*** (–3.69, –2.73)	–4.78*** (–5.48, –4.08)	–4.20*** (–4.69, –3.69)	–3.17*** (–3.90, –2.44)

APC, annual percent change; CI, confidence intervals.

* p–value < 0.05, ** p–value < 0.01, *** p–value < 0.001.

Table 2 shows the results of regression analysis investigating the interaction between marital status and year by sex and age group. The rate ratio for the interaction term between never-married persons and year was significantly higher than 1 among men aged 20–39 years, with the mortality rate ratio of 1.01 (95% CI:1.00, 1.02), while those were significantly lower than 1 among men aged 40–64 and ≥65 years, with the mortality rate ratios of 0.99 (95% CI:0.99, 1.00) and 0.98 (95% CI:0.98, 0.99), respectively. For women, the interactions term between never-married persons and year was not significant among persons aged 20–39 years, with the mortality rate ratio of 1.00 (95% CI:1.00, 1.01), while those were significantly lower than 1 among persons aged 40–64 and ≥65 years, with the mortality rate ratios of 0.98 (95% CI:0.98, 0.99) and 0.98 (95% CI:0.97, 0.99), respectively.

**Table 2:** Results of regression analysis investigating the interaction between marital status and year by sex and age group.

	20 − 39 years	40 − 64 years	≥65 years
	Rate ratio (95% CI)†	Rate ratio (95% CI)†	Rate ratio (95% CI)†
Men			
Year	0.98*** (0.98, 0.99)	0.95*** (0.95, 0.96)	0.97*** (0.96, 0.97)
Marital status			
Married	Reference	Reference	Reference
Never–married	2.63*** (2.45, 2.81)	2.53*** (2.36, 2.71)	2.94*** (2.68, 3.22)
Widowed	12.68*** (10.58, 15.20)	3.14*** (2.89, 3.41)	2.28*** (2.10, 2.47)
Divorced	12.32*** (10.99, 13.82)	5.92*** (5.43, 6.46)	4.31*** (3.89, 4.77)
Marital status × year			
Married × year	Reference	Reference	Reference
Never–married × year	1.01*** (1.00, 1.02)	0.99* (0.99, 1.00)	0.98*** (0.97, 0.99)
Widowed × year	0.98 (0.96, 1.00)	1.00 (0.99, 1.01)	0.99 (0.99, 1.00)
Divorced × year	0.99 (0.98, 1.00)	0.99 (0.99, 1.00)	0.98*** (0.98, 0.99)
Women			
Year	0.99*** (0.98, 0.99)	0.98*** (0.97, 0.98)	0.97*** (0.96, 0.97)
Marital status			
Married	Reference	Reference	Reference
Never–married	2.69*** (2.48, 2.92)	2.54*** (2.38, 2.72)	1.68*** (1.54, 1.84)
Widowed	7.31*** (5.40, 9.90)	2.21*** (2.04, 2.39)	1.61*** (1.49, 1.74)
Divorced	6.26*** (5.52, 7.10)	2.89*** (2.68, 3.10)	1.96*** (1.80, 2.14)
Marital status × year			
Married × year	Reference	Reference	Reference
Never–married × year	1.00 (1.00, 1.01)	0.98*** (0.98, 0.99)	0.98*** (0.97, 0.99)
Widowed × year	1.01 (0.98, 1.03)	0.99 (0.98, 1.00)	0.99** (0.98, 1.00)
Divorced × year	1.01 (1.00, 1.02)	1.00 (0.99, 1.01)	1.00 (0.99, 1.01)
CI, confidence intervals			

* p–value < 0.05, ** p–value < 0.01, *** p–value < 0.001.

†The five–year age groups, marital status, year (0–20), and an interaction term between marital status and year were included as the explanatory variables.

**Fig 6:**
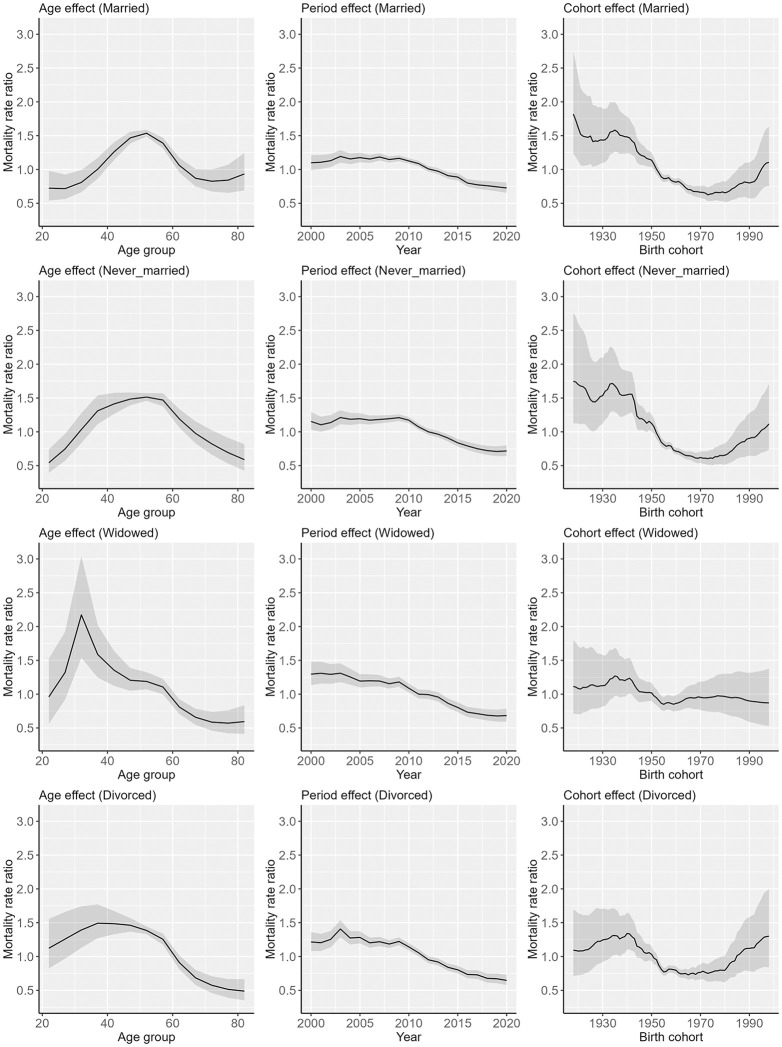
Results of age-period-cohort analysis by marital status for men. The solid line indicates the point estimation, and the shaded areas indicate 95% credible intervals.

Fig 6 presents the results of the age–period–cohort analysis by marital status for men, and S6 Table presents the underlying data in Fig 6. Mortality rate ratios were highest in middle-aged men among married and never-married men, while those peaked at younger ages among widowed and divorced men. A decreasing trend in the mortality rate ratio was noted for cohorts born between approximatley 1920 and 1970 among married and never-married men, while this trend was less pronounced among widowed and divorced men. In addition, an increasing trend in the mortality rate ratio was observed for cohorts born approximately 1970 or later among married, never-married, and divorced men, but not among widowed men.

**Fig 7:**
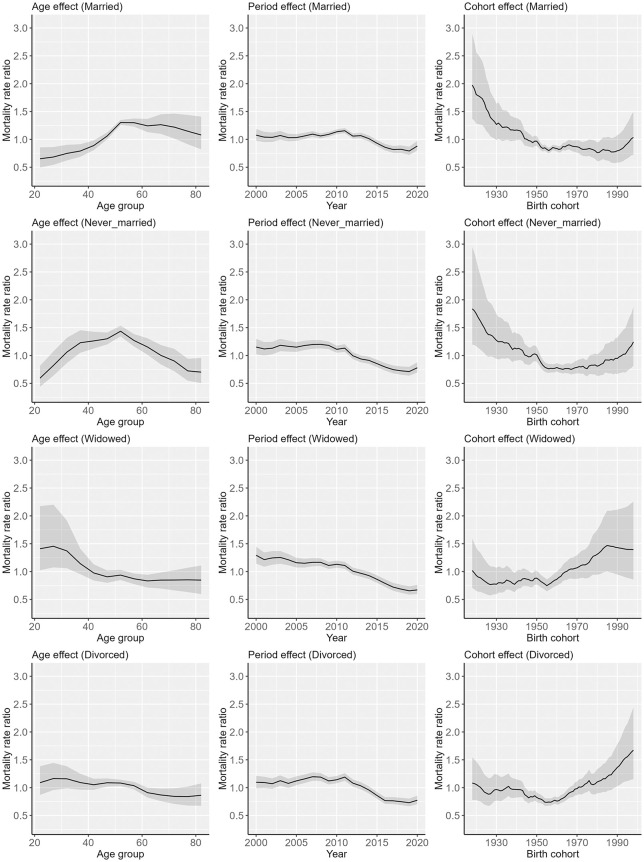
Results of age-period-cohort analysis by marital status for women. The solid line indicates the point estimation, and the shaded areas indicate 95% credible intervals.

Fig 7 shows the results of age-period-cohort analysis by marital status for women. Data from Fig 7 are shown in S7 Table. A similar result was observed to that in men for the age effect. A decreasing trend in the mortality rate ratio was observed for cohorts born between approximatley 1920 and 1960 for married and never-married women, while the decreasing trend was not evident among widowed and divorced women. In addition, an increasing trend in the mortality rate ratio was evident for cohorts born in the 1950s or later for widowed and divorced women and for cohorts born in the 1970s or later for never-married women, while the increasing trend was not evident in married women.

## Discussion

We examined the trends in suicide mortality rates by marital status in Japan. While married persons tended to exhibit the lowest suicide mortality rates across all age groups and years, the degree of change in these rates varied depending on the combination of marital status and age group. In this section, we discuss the potential causes and implications of these findings.

The rate ratios of the ASMRs for widowed and divorced persons relative to those for married persons varied over time, while these rate ratios tended to be higher at younger ages for both men and women regardless of year. In addition, the age–period–cohort analysis indicated that the peak ages for suicide differed between married persons and widowed and divorced persons. A study in Hong Kong found that the mortality rate ratio for divorced or separated persons compared with married persons was especially high among younger women [8], and a similar trend was observed among men and women in a South Korean study [14]. Psychological distress may play a role in these trends. A Norwegian study and a multinational study found that persons experiencing psychological distress and those with mental disorders were more likely to divorce [34,35], and a study in Canada showed that separated or divorced persons who were non-depressed tended to subsequently experience major depression [36]. In addition, another study in Canada showed that the association between depression and marital status differed depending on age, with the association being weaker for older persons than for younger persons [37].

Regarding widowed persons, a U.S. study indicated that suicide mortality tends to be especially high among younger widowed persons [9]. In addition, a South Korean study found that the suicide risk among widowed persons was significantly higher when their spouse had died by suicide, compared to other causes [38]. Given that suicide is a leading cause of death among younger adults, it is plausible that younger widowed persons are more likely to have lost their partner to suicide, thereby increasing their own risk. Moreover, a Canadian study showed that the odds of depression among widowed persons decreased with age [37], further supporting the possibility that age moderates the mental health consequences of marital dissolution.

Our results also showed that among men aged 20–39 years, the degree of decrease in suicide mortality rate for widowed persons was the largest. It has been noted that younger widows experienced more acute grief than older widows [9]. In Japan, grief care support for bereaved persons by private organizations has spread over decades, which may have contributed to reduced levels of grief and lower suicide mortality rates in this demographic over time. In contrast, among persons aged 40–64 years and ≥65 years, the most significant decrease in the suicide mortality rate from 2000 to 2020 was observed in never-married persons, for both men and women. In the 20th century, when marriage rates were higher in Japan, persons with poor health were more likely to remain unmarried [39], a phenomenon known as the marriage selection effect [40]. However, as the proportion of never-married persons has increased in recent decades, it is pointed out in a previous study that the link between being unmarried and poor health has weakened in Japan [39]. However, the results also demonstrated that the magnitude of decline in suicide mortality rates for married persons was larger than that for married personsin men and women across the study period among persons aged 20–39 years, with a significant difference noted in men. Recent studies have underscored an association between socioeconomic status and marital status in Japan [21,41], and differences in the proportion of married persons by education level increased over time during the 2010s in Japan [41]. Therefore, it is consiered that socioeconomic disparities in marital status appear to have increased among younger persons over the decades, which may have led to the results.

A particularly concerning finding was that significant downward trends were not observed for widowed and divorced women, whose suicide mortality rates were particularly high among women. The age–period–cohort analysis indicated increasing birth cohort effects of suicide mortality risk, especially among widowed and divorced women in recently born cohorts. In Japan, lower educational attainment was associated with higher divorce rates [42], and post-divorce income levels tended to decline significantly for women [43]. One study found that young women who recently experienced divorce faced reduced household income for up to 10 years, and the financial losses became larger in women who were divorced in recent years [44]. These worsening socioeconomic conditions may be related to the trend in suicide mortality rate among divorced young women in recent years. In addition, the suicide mortality rate for divorced young men was consistently higher than divorced young women. Moreover, suicide mortality rates were persistently higher among widowed and divorced men who were middle-aged or elderly than among their female counterparts over the decades. A study conducted in Canada reported that the association between depression and marital status was weaker for women than for men [37]. Therefore, it remains possible that the prevalence of depression was consistently higher among widowed and divorced men who were middle-aged or elderly than among similarly situated women in Japan as well.

It is important to pay close attention to the trend of suicide mortality among young persons in the future. In addition, the results suggest that expanded public support and social welfare programs targeting divorced and widowed persons who are young are necessary. Specifically, in addition to financial supports for persons with low income, social support easing psychological distress of divorced and widowed persons in younger ages is considered to be needed. Furthermore, examining long-term trends in psychiatric illness and sociodemographic profiles by marital status could help identify the root causes of this disparity. In addition, a future study using data from subsequent years needs to be conducted in order to verify whether the trends changes or not.

The strength of this study is that the national data in Japan over a long period of time were used and the results represent the overall trend in suicide mortality rate in Japan. In contrast, there are some limitations. A limitation of this study was that the information of persons was restricted to sex, age, marital status, and year because only those data were publicly available. Future studies should incorporate additional sociodemographic variables, such as occupation, educational attainment, regions, psychiatric distress, and living arrangements to provide a more comprehensive understanding of suicide risk factors. In addition, it is meaningful to survey the trend of sociodemographic characteristics of persons by marital status and age group in future studies. Moreover, estimated values were used for population size in years when the Census was not conducted. In addition, the possibility of misclassification and underreporting of suicide as a cause of death cannot be ruled out [45]. In contrast, one study reported that the frequency of suicides was not associated with the frequency of deaths due to unknown intent or causes in Japanese prefectures [46], indicating that suicides were unlikely to be misclassified as deaths of unknown intent or cause in Japan. Furthermore, the Census data on the married population included non-married persons in *de facto* relationships, whereas such persons were not classified as married in the Vital Statistics data. According to a survey conducted by the Cabinet Office of Japan, the proportion of persons in *de facto* relationships, relative to the combined number of those in *de facto* relationships and legally married persons, was approximately 3%–4% [47]. This discrepancy may have led to an underestimation of suicide mortality rates among married persons.

## Conclusions

Overall, a significant decreasing trend in suicide mortality rates from 2000 to 2020 was observed across all marital status groups among middle-aged and older persons. However, a significant decreasing trend in suicide mortality rates was not observed among never-married men aged 20–39 years and widowed and divorced women aged 20–39 years. In addition, over the decades, the rate ratios of ASMRs for widowed and divorced persons relative to those for married persons tended to be higher among younger age groups. Moreover, the age–period–cohort analysis revealed that mortality risk increased in more recently born cohorts particularly among widowed and divorced persons for women. The results indicated that trends in suicide mortality rates by marital status differed according to age group and suggested a possible need for targeted suicide prevention efforts within widowed and divorced persons in younger ages.

## Supporting information

S1 Table Data from Fig 1. (XLSX)

S2 Table Data from Fig 2. (XLSX)

S3 Table Data from Fig 3. (XLSX)

S4 Table Data from Fig 4. (XLSX)

S5 Table Data from Fig 5. (XLSX)

S6 Table Data from Fig 6. (XLSX)

S7 Table Data from Fig 7. (XLSX)

S8 Table Dataset used in the analysis. (CSV)
